# Seasonal variations in bird selection pressure on prey colouration

**DOI:** 10.1007/s00442-021-04994-9

**Published:** 2021-07-28

**Authors:** Elena L. Zvereva, Mikhail V. Kozlov

**Affiliations:** Department of Biology, University of Turku, 20014 Turku, Finland

**Keywords:** Bird community, Naïve predators, Plasticine models, Aposematic colouration, Cryptic colouration

## Abstract

The direction and strength of selection for prey colouration by predators vary in space and time and depend on the composition of the predator community. We tested the hypothesis that bird selection pressure on prey colouration changes through the season due to changes in the proportion of naïve juvenile individuals in the bird community, because naïve and educated birds differ in their responses to prey colours. Bird predation on caterpillar-shaped plasticine models in two boreal forest sites increased sevenfold from early summer to mid-summer, and the time of this increase coincides with the fledging of juvenile birds. In early summer, cryptic (black and green) models were attacked at fivefold higher rates compared with conspicuous (red and yellow) models. By contrast, starting from fledging time, cryptic and conspicuous models were attacked at similar rates, hinting at a lower selectivity by naïve juvenile birds compared with educated adult birds. Cryptic models exposed in a group together with conspicuous models were attacked by birds at a threefold lower rate than cryptic models exposed singly, thus supporting the aposematic commensalism hypothesis. However, this effect was not observed in mid- and late summer, presumably due to the lack of avoidance of conspicuous prey by the juvenile birds. We conclude that selection pressure on prey colouration weakens considerably when naïve birds dominate in the community, because the survival advantages of aposematic colouration are temporarily lost for both the conspicuous and their neighbouring cryptic prey.

**Supplementary Information:**

The online version contains supplementary material available at 10.1007/s00442-021-04994-9.

## Introduction

Birds use visual cues to find their prey, and therefore, bird predation is an important factor driving the evolution of colouration in various prey species (Lindstedt et al. 2011; Chouteau et al. 2016). Two major defensive strategies have evolved in organisms to reduce attacks by predators through colouration: crypsis, when prey avoids detection by adopting colours of their backgrounds, and aposematism, when prey is signalling about its defences through conspicuous colour patterns (Ruxton et al. 2018). The direction and strength of selection by predators for prey colouration are traditionally studied by observation of predator responses to particular colours and patterns in controlled experiments (reviewed by Ruxton et al. 2018). However, laboratory experiments have some limitations when extrapolated to natural conditions, because many environmental factors can affect the outcome of selection on prey colouration. In particular, natural variations in background characteristics and illumination can influence the responses of individual predators to prey colouration (Endler 1993; Rojas et al. 2014; Théry and Gomez 2010), potentially leading to spatial variations in selection pressure.

The direction of selection pressure may also differ between localities, depending on the species composition of the bird community (Nokelainen et al. 2014). In addition, it may differ within a locality, because bird communities change seasonally with respect to the proportion of naïve juvenile birds (Mappes et al. 2014). Adult birds generally avoid aposematic colours and patterns, because they have memorised previous unpleasant experiences with chemically defended or unpalatable prey usually possessing conspicuous colouration (Stevens and Ruxton 2012; Skelhorn et al. 2016). By contrast, juvenile birds have no such experience and usually must learn to avoid unpalatable prey based on colouration, although unlearnt biases against certain colours can also exist (Lindström et al. 1999; Halpin et al. 2020). Therefore, the behaviour of naïve juvenile birds in relation to prey colouration differs considerably from the behaviour of adult birds (Halpin et al. 2008; Svádová et al. 2013). These differences between naïve and educated birds are well known from laboratory experiments (Exnerová et al. 2007; Zvereva et al. 2018; Doktorovová et al. 2019), but surprisingly few studies have explored the consequences of these differences in the natural setting.

The changes in the proportion of naïve birds when the nestlings fledge and start feeding independently are ubiquitous. Therefore, studies comparing bird predation on prey of different colouration between seasons dominated by experienced versus naïve birds are badly needed. Studies with this focus will supplement the knowledge obtained in previous laboratory experiments and thus improve our understanding of the dynamics and overall direction of bird selection pressure on insect prey occurring in nature. Only a few studies have explicitly addressed this question (Mappes et al. 2014; Hernández-Agüero et al. 2020). In particular, Mappes et al. (2014) reported that an aposematic signal improves prey survival only in the early and late summer periods, when educated birds dominate the community. By contrast, at the naïvety peak, when young birds have just fledged, the conspicuousness of warning colours becomes a disadvantage to the prey. This previous study (Mappes et al. 2014) was conducted with plasticine model caterpillars, but investigated only one prey type with either cryptic (black) or aposematic (black with red spot) colourations. However, the responses of birds to aposematic prey may greatly depend on the type or pattern of the warning colouration (e.g., Nokelainen et al. 2014; Doktorovová et al. 2019). Therefore, further experiments are needed with models of different colours to generalise the initial findings of Mappes et al. (2014) to broader conditions.

Another phenomenon that can contribute to the evolution of prey colouration is a reduction in predation risk for palatable cryptic prey due to the proximity of aposematic prey (Mappes et al. 1999; de Wert et al. 2012). This benefit, termed aposematic commensalism, has been observed even in the absence of chemical defences, when both cryptic and aposematic objects (seeds) were palatable (de Wert et al. 2012). If the efficacy of an aposematic signal changes through the season depending on the proportion of naïve birds in the bird community (Mappes et al. 2014), then the expression of aposematic commensalism would also demonstrate seasonal variation.

The ultimate goal of the present study was to test the hypothesis that the strength and the direction of selective pressure by birds on the colouration of their insect prey vary within the season. In particular, we predicted that (i) aposematic colouration is advantageous for prey in spring and early summer, when the bird community consists of educated birds that avoid prey with aposematic colours; (ii) in mid-summer, when naïve juvenile birds fledge, the warning colouration becomes a disadvantage due to its high conspicuousness, in combination with the lack of aposematic colour avoidance by naïve fledglings; (iii) in late summer, when the juvenile birds have obtained experience with various natural prey, the aposematic colours again become advantageous; (iv) attack rates are lower for cryptic models than for aposematic models at fledgling time because the cryptic models are less conspicuous; and (v) cryptic prey benefit from their proximity to conspicuous prey when bird community consists of educated birds, but lose this benefit when bird community is dominated by naïve juvenile birds. We tested these predictions in field using prey models of two cryptic and two aposematic colours to account for natural prey variations.

## Methods

### Study sites and observations on birds

The experiments were conducted in 2020 at two sites located 58 km apart in southwestern Finland: one near Kustavi (60°31′58" N, 21°18′08" E) and the other near Turku (60°32′11" N, 22°21′52" E). The sites were managed Scots pine (*Pinus sylvestris*) forests with abundant understorey vegetation dominated by birches (*Betula pendula* and *B. pubescens*) and rowans (*Sorbus aucuparia*). At the first site, in March 2020, we placed three nest boxes about 100 m apart. By the end of May, these nest boxes were occupied by great tits (*Parus major*), blue tits (*Cyanistes caeruleus*), and pied flycatchers (*Ficedula hypoleuca*). Weekly observations of the birds that occupied these nest boxes allowed us to record bird phenology, including the construction of nests, feeding of nestlings, and time of fledging. Parent tits were seen visiting nests with food, and nestling calls were heard from 6 to 20 June. The parent visits of pied flycatcher continued until 28 June. We also observed several more bird species that possibly have contributed to the attacks on our models: *Erithacus rubecula, Fringilla coelebs, Phylloscopus trochilus, Emberiza citrinella, Sylvia borin, Turdus merula, Turdus iliacus*, and *Muscicapa striata*.

### Predation measurements

Plasticine models are increasingly used to measure predation pressure on herbivorous insect prey (Low et al., 2014; Roslin et al., 2017) in natural environments. Despite growing criticism of this method (Lövei and Ferrante 2017; Zverev et al. 2020), researchers generally accept that the attack rates on plasticine models—although they do not reflect the absolute values of predation pressure—can be useful in comparisons between different localities (Tvardikova and Novotny 2012; Roslin et al. 2017; Zvereva et al. 2019). Therefore, we have assumed that this method is suitable for comparisons of bird predation on prey of different colours through the season.

In this study, we used prey models of four colours: black and green were classified as cryptic, and red and yellow as conspicuous (aposematic) colours. Both red and yellow are long-wavelength colours, which are known to provide effective aposematic signals (Stevens and Ruxton 2012). Green is a typical cryptic colour for herbivorous insects (Aslam et al. 2020), while black may be considered differently. In some experiments, black food was least preferred by chicks (Roper and Marples 1997), but we follow studies using plasticine models in natural conditions (Mappes et al. 2014; Zvereva et al. 2019) which considered black as cryptic colour. We also conducted a test for the relative conspicuousness/crypsis of colours used in our experiment (see below).

We fabricated caterpillar-shaped models (25–30 mm length and 4–5 mm diameter) from non-toxic odourless modelling coloured clay (Chemical plant ‘Luch’, Yaroslavl, Russia) and used wire 0.3 mm in diameter to attach the models to thin branches in the outer parts of tree crowns at a height of 1.2–1.8 m. In each site, we haphazardly selected 25 mature (> 2 m height) birches (*B. pubescens*) and 25 pines (2–3 m height) arranged in five blocks located > 50 m apart. Each block consisted of five trees of each species growing at least 5 m apart. In each block, we attached, in a haphazard order, four models of different colours (at about 20 cm apart) to one birch and one pine, and one model of each of colours to four birches and four pines, which resulted in 16 models per block and 80 models per site. In Kustavi the experiment started on 17-May-2020 and lasted until 20-Sep-2020; in Turku, it started on 22-May-2020 and lasted until 24-Sep-2020. Records were collected at 1-week intervals resulting in 18 records.

Beak marks left by birds on the prey models were identified according to Low et al. (2014) and counted (Online resource 1). The attacked models were then either remoulded or replaced if the damage was severe. When a model (including the wire) was not found, this record was considered as missing (because the reasons for the model disappearance were unclear), and a new model was placed on the tree.

Similar to our previous study (Zvereva et al. 2019), we used two variables to characterize bird predation on plasticine models: (1) the proportion of the attacked models (rate of attacks hereafter; this variable can be interpreted as the prey mortality rate) and (2) number of beak marks on each attacked model (intensity of attacks hereafter). As the records were collected at 1-week intervals, the multiple beak marks observed on our models could reflect either multiple pecks made during a single attack or several attacks divided over time. However, in both situations, repeated attacks on a model reflect model’s attractiveness for the birds.

During each count of bird attacks on our models, on trees with four models, we recorded the sequence in which models of different colours were found by the observer (ELZ). This sequence served as a score for each model (Online resource 1), with the lowest score (1) corresponding to the model that had been found first (i.e., was most conspicuous). The mean scores were then compared among colours. Although vision differs in birds and humans, the detectability of an object by the human eye is frequently extrapolated to natural predators (Bohlin et al. 2012; Mappes et al. 2014; Karpestam et al. 2018).

### Data analysis

To address seasonal variation in bird selection pressure on prey colouration, we divided the growing season into three periods based on our own observations and accounting for the data provided by Mappes et al. (2014) for southern Finland. During the first period (from mid-May to the third week of June; early summer hereafter), the bird population consisted of adult birds that were constructing their nests, laying eggs, and then feeding their nestlings. The second period (from the end of June to the beginning of August; mid-summer hereafter) started when the juvenile birds left their nests and tried to search for food on their own. At this time, parent birds discontinued their visits to nest boxes, and the bird population became dominated by young naïve birds. During the third period (August to mid-September; late summer hereafter), the young birds were expected to have gained experience in foraging for various natural prey (Mappes et al. 2014) and would therefore no longer be naïve to prey with warning colouration.

We used a mixed model ANOVA (SAS GLIMMIX procedure, type III tests; SAS Institute 2009) with different model statements: binomial distribution with logit link function for the attack rate, Poisson distribution with log link function for the attack intensity, and Gaussian distribution for the order in which models were found by the observer (averaged across the five conspecific trees at each site for each census). In all these analyses, we considered site (Kustavi or Turku), period (early, mid-, and late summer), signal (cryptic or conspicuous), colour nested within signal, tree species (birch or pine), grouping (multi-colour group or single model) and their interactions as fixed effects, and block nested within site as a random effect. We facilitated accurate *F* tests of the fixed effects by adjusting the standard errors and denominator degrees of freedom using the latest version of the method by Kenward and Roger (2009). The significance of random effects was explored by a likelihood ratio test (Stroup 2013).

## Results

### Spatial variation in bird predation

The between-site difference in attack rates was marginally significant (Table 1). The proportion of models attacked during 1 week was slightly higher in Kustavi (where nest boxes were established) than in Turku (mean ± SE: 0.38 ± 0.033 and 0.28 ± 0.028, respectively). Variation among the blocks within each site was highly significant, indicating high small-scale spatial variations in bird predation (Table 1). The models placed on birches and pines were attacked at similar rates (Table 1).

**Table 1:** Sources of variation in bird attack characteristics on caterpillar-shaped plasticine models of four different colours (mixed model ANOVA, type III tests). Signal: aposematic or cryptic colouration; grouping: models of different colours exposed in mixed groups or singly; period: early, mid-, and late summer

Effect	Source of variation	Attack rate (proportion of models attacked during a week)		Attack intensity (number of beak marks on attacked model by the end of week)	
		Test statistics	*P* value	Test statistics	*P* value
Fixed	Site	*F*_1, 7.79_ = 4.57	0.07	*F*_1, 7.97_ = 1.55	0.25
Fixed	Signal	*F*_1, 2860_ = 17.44	< 0.0001	*F*_1, 774_ = 0.49	0.48
Fixed	Colour (signal)	*F*_2, 2860_ = 24.26	< 0.0001	*F*_2, 774_ = 5.20	0.0057
Fixed	Period	*F*_2, 2860_ = 57.56	< 0.0001	*F*_2, 774_ = 9.93	< 0.0001
Fixed	Tree species	*F*_1, 2860_ = 1.82	0.18	*F*_1, 774_ = 1.63	0.20
Fixed	Grouping	*F*_1, 2860_ = 2.44	0.12	*F*_1, 774_ = 1.65	0.20
Fixed	Signal × tree species	*F*_1, 2860_ = 0.07	0.79	*F*_1, 774_ = 7.40	0.0067
Fixed	Signal × period	*F*_2, 2860_ = 6.84	0.0011	*F*_2, 774_ = 1.83	0.16
Fixed	Signal × grouping	*F*_1, 2860_ = 0.15	0.70	*F*_1, 774_ = 1.95	0.16
Fixed	Period × tree species	*F*_2, 2860_ = 1.05	0.35	*F*_2, 774_ = 0.03	0.97
Fixed	Period × grouping	*F*_2, 2860_ = 2.90	0.05	*F*_2, 774_ = 3.21	0.0410
Fixed	Grouping × tree species	*F*_1, 2860_ = 7.75	0.0054	*F*_1, 774_ = 0.33	0.57
Random	Block (site)	*χ*^2^_1_ = 15.6	< 0.0001	*χ*^2^_1_ = 26.4	< 0.0001

### Temporal variation in bird predation

**Fig. 1:**
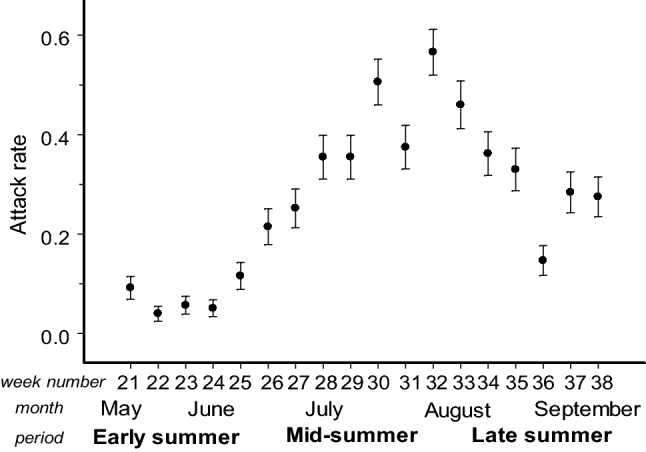
Seasonal changes in bird attack rates on plasticine models (all colours combined). Values are estimated marginal means ± SE for proportion of models attacked during one week

**Fig. 2:**
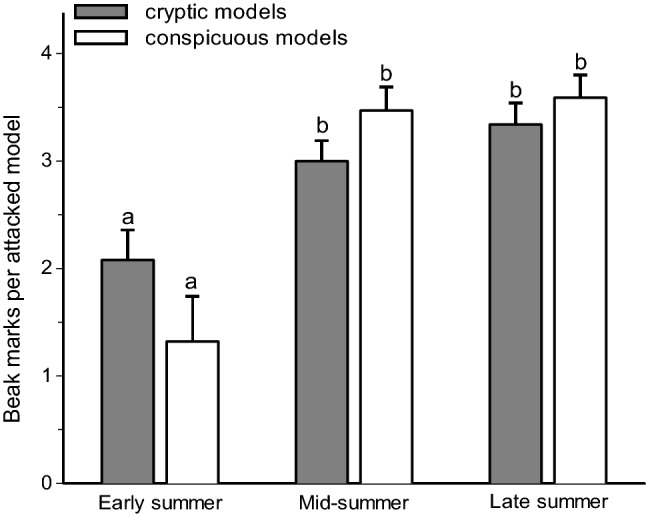
Seasonal changes in the intensities of bird attacks on plasticine models of cryptic (black and green) and aposematic (red and yellow) colours. Values are estimated marginal means + SE. Bars marked with different letters significantly differ from each other (mixed model ANOVA, *t* test)

**Fig. 3:**
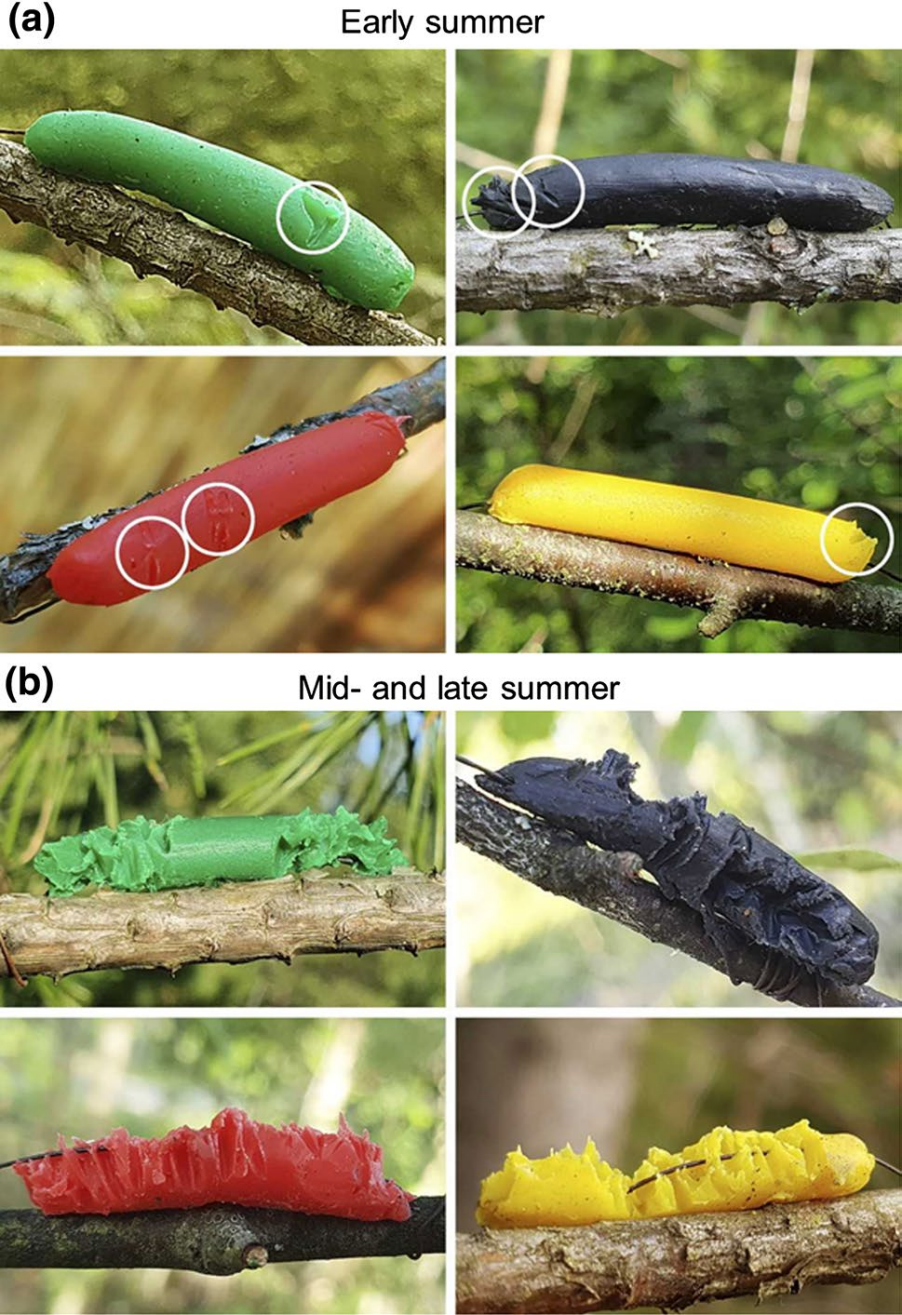
Bird beak marks on plasticine models typical for early summer (**a**) and for mid- and late summer (**b**). Circles denote locations of weak beak marks

Attack rates on plasticine models significantly differed between the observation periods (Table 1): they were lowest in early summer, started to increase during the third week of June, and then remained high through mid- and late summer, followed by a decrease in September (Fig. 1). On average, the attack rates in mid- and late summer were seven times higher than those in early summer. The attack intensity (number of beak marks per attacked model) was also lowest in early summer and remained high during mid- and late summer (Table 1, Figs. 2, 3).

### Effect of model colouration on bird predation

**Fig. 4:**
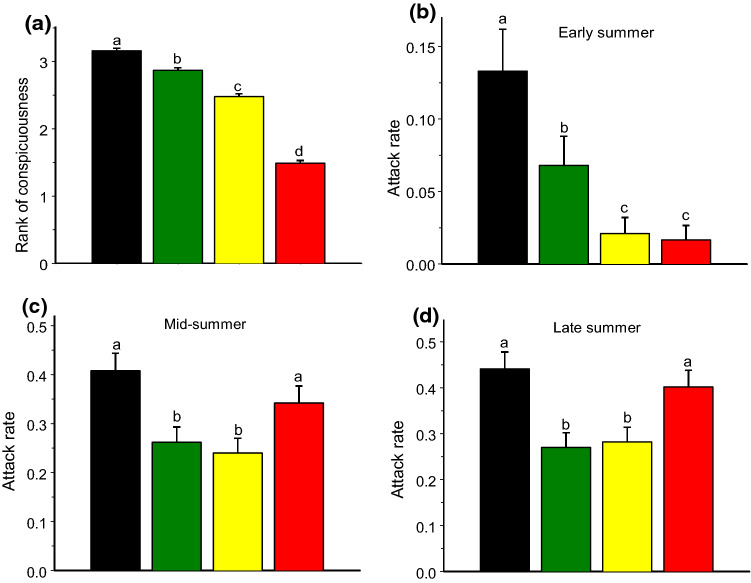
Conspicuousness of colours used in the experiment for human eye (higher rank corresponds to lower conspicuousness) (**a**) and bird attack rates on models of these colour in early (**b**), mid- (**c**), and late (**d**) summer. Values are estimated marginal means + SE for proportion of models attacked during 1 week. Bars marked with different letters significantly differ from each other (mixed model ANOVA, *t* test)

**Fig. 5:**
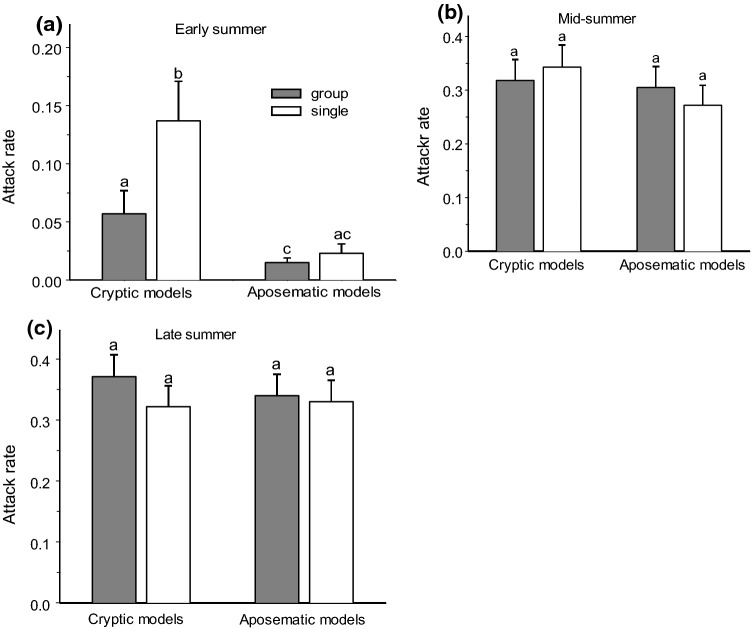
Bird attack rates on cryptic (black and green) and aposematic (red and yellow) models offered in mixed groups of all four colours and singly in a tree in different periods of season: early summer (**a**), mid-summer (**b**), and late summer (**c**). Values are estimated marginal means + SE for proportion of models attacked during 1 week. Bars marked with different letters significantly differ from each other (mixed model ANOVA, *t* test)

The prey conspicuousness differed among colours (*F*_3, 112_ = 281.7, *p* < 0.0001): red models were the most conspicuous and black models were the most cryptic (Fig. 4a). The probability of being attacked was also highly affected by the model colour (Table 1). In early summer, the attack rates closely followed the rank of conspicuousness, decreasing from the most cryptic to the most conspicuous model (Fig. 4b). As a result, the cryptic (black and green) models were attacked five times more frequently than the conspicuous (red and yellow) models (Fig. 5a). However, this difference disappeared in mid-summer (Fig. 5b, c). Within the cryptic colours, the black models were attacked at greater rates than the green models throughout the entire season, while within the aposematic colours, red models were attacked at greater rate than yellow models in mid- and late summer (Table 1; Fig. 4b, c).

### Effect of association of differently coloured prey on bird predation

The effects of grouping of cryptic and conspicuous models on bird attack rates differed between periods (Table 1). In early summer, cryptic models exposed jointly with conspicuous models were attacked by birds at a threefold lower rate than were models exposed singly on a tree (Fig. 5). However, this difference disappeared in the mid-summer (Fig. 5b, c). Attacks on conspicuous models did not depend on whether they were exposed jointly with cryptic models or singly (Fig. 5).

## Discussion

### Seasonal changes in bird predation on the insect models

Exploration of the seasonal changes in predation pressure necessarily involves exposures of artificial prey at the same site over several months. In the case of inedible plasticine models, this can potentially result in a decrease in bird attacks with time as, e.g., observed by Hernández-Agüero et al. (2020), because the birds might learn to avoid items which provide no nutritional reward. Nevertheless, many studies employing long-lasting exposure of models revealed no decreases in bird predation rates on plasticine prey over time (Lemessa et al. 2015; Mappes et al. 2014; Kozlov et al. 2017; Zvereva et al. 2019). These results indicate that avoidance learning of neutral stimuli (neither unpalatable nor rewarding), even if it occurs, is relatively slow (Alcock 1970a) and does not compromise the outcomes of long-term observations.

Only a handful of studies have explored seasonal changes in the intensity of bird predation on herbivorous insects, and the outcomes of these studies have been somewhat contradictory (Remmel et al. 2009; Mappes et al. 2014; Kozlov et al. 2017; Pan et al. 2020). In our study, predation peaked in mid-summer, and remained at a high level until late summer. These results are similar to those reported by Kozlov et al. (2017), who measured bird predation in seven forests situated at approximately the same latitude as our sites. The latter pattern may be explained by the appearance of juvenile birds, which fledge and start to feed independently in our geographical region beginning in late June (Mappes et al. 2014; our observations). Thus, the abundance of foraging birds increased greatly at that time.

However, the maximum value of attack rates during mid-summer exceeded the early summer attack rates by more than tenfold. Keeping in mind that the brood size of passerine birds is considerably lower than 18, which is the number that would be needed to reach a tenfold increase in predation rates, this large difference cannot emerge from the increase in bird numbers alone. This result could be explained by lower capacity of plasticine to be marked by bird attack at lower temperatures (Muchula et al. 2019). However, even in early summer, variation in daily temperatures were within the range for which Muchula et al. (2019) did not find any difference in the visibility of attack marks. The difference in attack rates between early and mid-summer also could not be explained by seasonal changes in day length, because in our study region, these changes were minor (from 18.2 h in early summer to 17.6 h in mid-summer). We therefore suggest that the low level of bird predation on model prey in the early summer is explained by the cautiousness of adult birds during encounters with artificial prey, as indicated by relatively weak beak marks on attacked models in early summer (Fig. 3a). This is because artificial prey do not present all required stimuli (Théry and Gomez 2010) and are generally attacked at a lower rate when compared with real prey (Lövei and Ferrante 2017). High cautiousness of adult birds in attacking models may also explain the difference between our results and the results by Remmel et al. (2009), who made prey models from fly puparia (i.e., these models were edible) and who observed the highest predation when bird community consisted of adult birds. At the same time, juvenile birds are more explorative than the adult birds and are therefore less cautious when attacking potential food items (Greenberg and Mettke-Hofmann 2001). These differences in the behaviour of adult and juvenile birds, together with the increase in bird numbers, could explain the observed higher rate of predation on artificial caterpillars in mid-summer than in early summer. The slight decrease in predation rates observed in late summer might be attributed to the mortality of birds (predominantly juveniles) from predation and/or to learning by juveniles to avoid non-rewarding items (Hernández-Agüero et al. 2020). Furthermore, in September, some migratory species (e.g., *Ficedula hypoleuca*, *Fringilla coelebs, *and* Erithacus rubecula*) already started departing to their wintering grounds.

### Seasonal variation in bird attacks on prey of different colours

We attribute the seasonal variation in the distribution of bird attacks on prey of different colouration to changes in the proportion of naïve juvenile individuals in the bird community. In the early summer, when the bird community consisted of adult educated birds, the prey with conspicuous colours were attacked considerably less frequently than the cryptic prey. Later in the season, when the nestlings had fledged and began searching for food, both the frequency and the intensity of the attacks increased substantially on the models with conspicuous colouration. Attacks on aposematic prey increased by 16-fold from early to mid-summer, whereas attacks on cryptic prey increased only threefold. This observation is in line with the results of Mappes et al. (2014), who also found that aposematic colouration does not provide any considerable survival advantage to insect prey during the naïvety peak in mid-summer. However, contrary to our predictions, we did not find any increased advantage of cryptic colouration when the young birds started their independent feeding: the attacks were equally as frequent and intense on the cryptically coloured models as on the conspicuous models (Fig. 5b, c).

The low selectivity of bird attacks in relation to prey colouration in mid-summer could be explained by the exploratory behaviour of the juvenile birds. Juvenile birds are more exploratory than adult birds, because they begin life with no information about their environment; therefore, the potential benefits of exploration are great. The mid-summer increase in the attack intensity may be also attributed to this exploratory behaviour of juvenile birds, as the juveniles pecked the attacked model many times to check whether the attacked item was edible (Fig. 3b). By contrast, the benefits of exploration are considerably reduced in adult birds, and these birds have generally higher levels of neophobia compared to juvenile birds (Greenberg and Mettke-Hofmann 2001). The stronger colour preferences of adult birds compared with fledglings may be also explained by the older birds’ previous experiences with defended, warningly coloured prey, e.g., yellow-coloured wasps and red-coloured leaf beetles *Chrysomela populi* and ladybirds, which were common in both study areas, while juvenile birds have not yet had this type of experience. The similar frequency and intensity of attacks on cryptic and aposematic models in mid-summer may be explained by the coexistence of adult educated birds, which preferentially attacked cryptic models, and juvenile naïve birds, which preferentially attacked conspicuous models. The end result was that the conspicuous and cryptic prey were attacked at the same rates.

Based on a study by Mappes et al. (2014), we expected to find that aposematic models would be avoided in late summer, once the juvenile birds had obtained the experience with natural aposematic prey. However, we did not observe any decrease in bird attacks on the aposematic models in late summer compared with mid-summer. This difference between our study and that of Mappes et al. (2014) could not be explained by the activity of naïve juvenile birds from the second clutches, because none of the nest boxes in the Kustavi site were occupied for the second time. We also have to exclude the hypothesis that, in late summer, a red colour becomes a signal of profitability of the fruits that are food for omnivorous birds, because birds can distinguish the shape of red objects and avoid them when they are insect-shaped (Gamberale-Stille et al. 2007). We therefore suggest that, in our study, learning to avoid our models could be slow or even non-existent, because they neither provided any reward nor contained noxious chemicals (Alcock 1970a, b), while experience with natural aposematic prey, which appearance could be generalised, was limited.

The colours typical of aposematic prey patterns facilitate the avoidance learning of unpalatable prey (Ruxton et al. 2018), but the bias against some colour patterns may be innate (Roper and Cook 1989; Hauglund et al. 2006; Pegram and Rutowski 2014). We did not observe any avoidance of red models when the bird community was dominated by juvenile birds, indicating the absence of an innate avoidance of red. However, yellow models were attacked by juvenile birds at significantly lower rates compared to red models. Some studies showed that, among aposematic colours, yellow was more effective for avoidance learning than red (Lawrence and Noonan 2018), while in other studies, birds learned to avoid the red moth models considerably faster than the yellow models (Rönkä et al. 2018). We suggest that in our study, this difference between attacks on yellow and red models may be explained by the innate nature of aversive responses to yellow prey, as indicated by some experiments (Lindström et al. 1999; Hauglund et al. 2006). In general, birds usually show stronger innate avoidance responses to brightly coloured than to dull-coloured foods (Greenberg and Mettke-Hofmann 2001), and our yellow models had higher luminance compared with our red models (Zvereva et al. 2019). Thus, our study shows that yellow models cause innate avoidance, whereas aversion to red has to be learned by birds.

Our study has added to the limited knowledge on the relative efficacy of two major strategies of two types of adaptive coloration that reduce the risk of predation. Some earlier experiments showed better survival of natural aposematic prey compared with cryptic prey (Sillén-Tullberg 1985; Aslam et al. 2020), but the relative efficacy of cryptic and aposematic colouration may depend on the environment (Seymoure et al. 2018). We found that the relative efficacy of these two strategies also depends on the composition of the bird community with respect to the naïvety level. Adult birds easily find cryptic prey while avoiding aposematic prey, whereas none of these strategies seem to provide survival benefits in juvenile naïve birds due to their high exploratory activity.

### Aposematic commensalism

A close proximity to aposematic prey may affect the fate of cryptic prey (de Wert et al. 2012). Cryptic prey would suffer greater predation if the conspicuous prey draws the attention of predators to the cryptic prey’s location. Alternatively, cryptic prey may benefit from proximity to their aposematic neighbours, because the bird predation risk could be reduced through aposematic commensalism (Mappes et al. 1999; de Wert et al. 2012). Our study supports the latter hypothesis, because we demonstrated a lower bird attack frequency on cryptic models exposed in a group that included aposematic models.

An important point is that aposematic commensalism was observed in the case of artificial models that lacked any taste stimuli, indicating that colouration of aposematic prey rather than defence is the most important cue in providing survival benefits for cryptic prey. These benefits might occur if predators avoid locations where they notice aposematically coloured prey, as the predators have previously learned that these are noxious. This phenomenon may have potential consequences for the evolution of Batesian mimicry, as discussed by de Wert et al. (2012). However, our results clearly show that aposematic commensalism in nature might only be observed when aposematic prey are avoided by the majority of birds, i.e., when the bird community is dominated by educated birds. When aposematic prey colouration is not avoided, as in the case of naïve birds, cryptic prey does not obtain any benefits from grouping with aposematic prey. Thus, the seasonal shift in the bird community from a domination by educated birds to a domination by naïve birds changes the selection pressure on prey colouration both through increased predation on aposematic prey and through loss of the survival benefits obtained by cryptic prey due to proximity of aposematic prey.

We conclude that selection pressure on prey colouration weakens considerably when naïve birds dominate in the community. This is because the survival advantages of aposematic colouration are temporarily lost for both conspicuous prey and their neighbouring cryptic prey. These changes may have important consequences for within-season variations in insect prey colouration. From a methodological perspective, our results show that the age composition of the bird community should be taken into account in field studies of bird selection pressures on prey colouration.

## Supplementary Information

Below is the link to the electronic supplementary material.

Supplementary file1 (XLS 465 kb)

## Data Availability

All data produced from this study are provided in the manuscript or in the Electronic Supplemental Material.

## References

[CR1] Alcock J (1970). Punishment levels and the response of black-capped chickadees (*Parus atricapillus*) to three kinds of artificial seeds. Anim Behav.

[CR2] Alcock J (1970). Punishment levels and response of white-throated sparrows (*Zonotrichia albicollis*) to three kinds of artificial models and mimics. Anim Behav.

[CR3] Aslam M, Nedvěd O, Sam K (2020). Attacks by predators on artificial cryptic and aposematic insect larvae. Entomol Exp Appl.

[CR4] Bohlin T, Gamberale-Stille G, Merilaita S, Exnerová A, Stys P, Tullberg BS (2012). The detectability of the colour pattern in the aposematic firebug, *Pyrrhocoris apterus*: an image-based experiment with human ‘predators’. Biol J Linn Soc.

[CR5] Chouteau M, Arias M, Joron M (2016). Warning signals are under positive frequency-dependent selection in nature. Proc Nat Acad Sci USA.

[CR6] de Wert L, Mahon K, Ruxton GD (2012). Protection by association: evidence for aposematic commensalism. Biol J Linn Soc.

[CR7] Doktorovová L, Exnerová A, Hotová Svádová K, Štys P, Adamová-Ježová D, Zverev V, Kozlov MV, Zvereva EL (2019). Differential bird responses to colour morphs of an aposematic leaf beetle may affect variation in morph frequencies in polymorphic prey populations. Evol Biol.

[CR8] Endler JA (1993). The color of light in forests and its implications. Ecol Monogr.

[CR9] Exnerová A, Štys P, Fucíková A, Vesela S, Svádová K, Prokopová M, Jarosik V, Fuchs R, Landová E (2007). Avoidance of aposematic prey in European tits (Paridae): learned or innate?. Behav Ecol.

[CR10] Gamberale-Stille G, Hall KSS, Tullberg BS (2007). Signals of profitability? Food colour preferences in migrating juvenile blackcaps differ for fruits and insects. Evol Ecol.

[CR11] Greenberg R, Mettke-Hofman C, Nolan V, Thompson CF (2001). Ecological aspects of neophobia and neophilia in birds. Current ornithology.

[CR12] Halpin CG, Skelhorn J, Rowe C (2008). Naïve predators and selection for rare conspicuous defended prey: the initial evolution of aposematism revisited. Anim Behav.

[CR13] Halpin CG, Penacchio O, Lovell PG, Cuthill IC, Harris JM, Skelhorn J, Rowe C (2020). Pattern contrast influences wariness in naïve predators towards aposematic patterns. Sci Rep.

[CR14] Hauglund K, Hagen SB, Lampe HM (2006). Responses of domestic chicks (*Gallus gallus domesticus*) to multimodal aposematic signals. Behav Ecol.

[CR15] Hernández-Agüero JA, Polo V, García M, Simón D, Ruiz-Tapiador I, Cayuela L (2020). Effects of prey colour on bird predation: an experiment in Mediterranean woodlands. Anim Behav.

[CR16] SAS Institute (2009) SAS/Stat. User's guide, version 9.2. SAS Institute, Cary

[CR17] Karpestam E, Merilaita S, Forsman A (2018). Size variability effects on visual detection are influenced by colour pattern and perceived size. Anim Behav.

[CR18] Kenward MG, Roger JH (2009). An improved approximation to the precision of fixed effects from restricted maximum likelihood. Comput Stat Data Anal.

[CR19] Kozlov MV, Lanta V, Zverev V, Rainio K, Kunavin MA, Zvereva EL (2017). Decreased losses of woody plant foliage to insects in large urban areas are explained by bird predation. Global Change Biol.

[CR20] Lawrence JP, Noonan BP (2018). Avian learning favors colorful, not bright, signals. PLoS ONE.

[CR21] Lemessa D, Hamback PA, Hylander K (2015). Arthropod but not bird predation in Ethiopian homegardens is higher in tree-poor than in tree-rich landscapes. PLoS ONE.

[CR22] Lindstedt C, Eager H, Ihalainen E, Kahilainen A, Stevens M, Mappes J (2011). Direction and strength of selection by predators for the color of the aposematic wood tiger moth. Behav Ecol.

[CR23] Lindström L, Alatalo RV, Mappes J (1999). Reactions of hand-reared and wild-caught predators toward warningly colored, gregarious, and conspicuous prey. Behav Ecol.

[CR24] Lövei GL, Ferrante M (2017). A review of the sentinel prey method as a way of quantifying invertebrate predation under field conditions. Insect Sci.

[CR25] Low PA, Sam K, McArthur C, Posa MRC, Hochuli DF (2014). Determining predator identity from attack marks left in model caterpillars: guidelines for best practice. Entomol Exp Appl.

[CR26] Mappes J, Tuomi J, Alatalo RV (1999). Do palatable prey benefit from aposematic neighbors?. Ecoscience.

[CR27] Mappes J, Kokko H, Ojala K, Lindström L (2014). Seasonal changes in predator community switch the direction of selection for prey defences. Nat Commun.

[CR28] Muchula K, Xie G, Gurr GM (2019). Ambient temperature affects the utility of plasticine caterpillar models as a tool to measure activity of predators across latitudinal and elevational gradients. Biol Control.

[CR29] Nokelainen O, Valkonen J, Lindstedt C, Mappes J (2014). Changes in predator community structure shifts the efficacy of two warning signals in arctiid moths. J Anim Ecol.

[CR30] Pan X, Mizuno T, Ito K, Ohsugi T, Nishimichi S, Nomiya R, Nomiya R, Ohno M, Yamawo A, Nakamura A (2020). Assessing temporal dynamics of predation and effectiveness of caterpillar visual defense using sawfly larval color and resting posture as a model. Insect Sci.

[CR31] Pegram KV, Rutowski RL (2014). Relative effectiveness of blue and orange warning colours in the contexts of innate avoidance, learning and generalization. Anim Behav.

[CR32] Remmel T, Tammaru T, Magi M (2009). Seasonal mortality trends in tree-feeding insects: a field experiment. Ecol Entomol.

[CR33] Rojas B, Rautiala P, Mappes J (2014). Differential detectability of polymorphic warning signals under varying light environments. Behav Proces.

[CR34] Rönkä K, De Pasqual C, Mappes J, Gordon S, Rojas B (2018). Colour alone matters: no predator generalization among morphs of an aposematic moth. Anim Behav.

[CR35] Roper TJ, Cook SE (1989). Responses of chicks to brightly colored insect prey. Behaviour.

[CR36] Roper TJ, Marples NM (1997). Colour preferences of domestic chicks in relation to food and water presentation. Appl Anim Behav Sci.

[CR37] Roslin T, Hardwick B, Novotny V, Petry WK, Andrew NR, Asmus A, Barrio IC, Basset Y, Boesing AL, Bonebrake TC, Cameron EK, Dattilo W, Donoso DA, Drozd P, Gray CL, Hik DS, Hill SJ, Hopkins T, Huang S, Koane B, Laird-Hopkins B, Laukkanen L, Lewis OT, Milne S, Mwesige I, Nakamura A, Nell CS, Nichols E, Prokurat A, Sam K, Schmidt NM, Slade A, Slade V, Suchankova A, Teder T, van Nouhuys S, Vandvik V, Weissflog A, Zhukovich V, Slade EM (2017). Higher predation risk for insect prey at low latitudes and elevations. Science.

[CR38] Ruxton GD, Allen WL, Sherratt TN, Speed MP (2018). Avoiding attack: the evolutionary ecology of crypsis, warning signals, and mimicry.

[CR39] Seymoure BM, Raymundo A, McGraw KJ, McMillan WO, Rutowski RL (2018). Environment-dependent attack rates of cryptic and aposematic butterflies. Current Zool.

[CR40] Sillén-Tullberg B (1985). Higher survival of an aposematic than of a cryptic form of a distasteful bug. Oecologia.

[CR41] Skelhorn J, Halpin CG, Rowe C (2016). Learning about aposematic prey. Behav Ecol.

[CR42] Stevens M, Ruxton GD (2012). Linking the evolution and form of warning coloration in nature. Proc Royal Soc B.

[CR43] Stroup WW (2013). Generalized linear mixed models: modern concepts, methods and applications.

[CR44] Svádová K, Exnerová A, Kopecková M, Štys P (2013). How do predators learn to recognize a mimetic complex: experiments with naïve great tits and aposematic Heteroptera. Ethology.

[CR45] Théry M, Gomez D, Casas J, Simpson SJ (2010). Insect colours and visual appearance in the eyes of their predators. Advances in insect physiology.

[CR46] Tvardikova K, Novotny V (2012). Predation on exposed and leaf-rolling artificial caterpillars in tropical forests of Papua New Guinea. J Trop Ecol.

[CR47] Zverev V, Zvereva EL, Kozlov MV (2020). Bird predation does not explain spatial variation in insect herbivory in a forest–tundra ecotone. Polar Biol.

[CR48] Zvereva EL, Doktorovová L, Hotová Svádová K, Zverev V, Štys P, Adamová-Ježová D, Kozlov MV, Exnerová A (2018). Defence strategies of *Chrysomela lapponica* (Coleoptera: Chrysomelidae) larvae: relative efficacy of secreted and stored defences against insect and avian predators. Biol J Linn Soc.

[CR49] Zvereva EL, Castagneyrol B, Cornelissen T, Forsman A, Hernández-Agüero JA, Klemola T, Paolucci L, Polo V, Salinas N, Theron KJ, Xu GR, Zverev V, Kozlov MV (2019). Opposite latitudinal patterns for bird and arthropod predation revealed in experiments with differently colored artificial prey. Ecol Evol.

